# Ordinary and Activated Bone Grafts: Applied Classification and the Main Features

**DOI:** 10.1155/2015/365050

**Published:** 2015-11-15

**Authors:** R. V. Deev, A. Y. Drobyshev, I. Y. Bozo, A. A. Isaev

**Affiliations:** ^1^Human Stem Cells Institute, Moscow 199333, Russia; ^2^Department of Morphology and General Pathology, Kazan (Volga Region) Federal University, Kazan 420008, Russia; ^3^Department of Maxillofacial and Plastic Surgery, A.I. Evdokimov Moscow State University of Medicine and Dentistry, Moscow 127473, Russia; ^4^Department of Maxillofacial Surgery, A.I. Burnasyan Federal Medical Biophysical Center, Moscow 123098, Russia

## Abstract

Bone grafts are medical devices that are in high demand in clinical practice for substitution of bone defects and recovery of atrophic bone regions. Based on the analysis of the modern groups of bone grafts, the particularities of their composition, the mechanisms of their biological effects, and their therapeutic indications, applicable classification was proposed that separates the bone substitutes into “ordinary” and “activated.” The main differential criterion is the presence of biologically active components in the material that are standardized by qualitative and quantitative parameters: growth factors, cells, or gene constructions encoding growth factors. The pronounced osteoinductive and (or) osteogenic properties of activated osteoplastic materials allow drawing upon their efficacy in the substitution of large bone defects.

## 1. Introduction

Bone grafting procedures are one of the main practice components in traumatology, orthopaedics, and maxillofacial surgery. The high rate of such surgeries is associated with the frequency and variety of pathological conditions that result in the formation of bone defects. The specific group of indications for osteoplastic materials in traumatology and orthopaedics consists of degenerative diseases of spine and major joints and, in dental and maxillofacial surgery, the atrophy of the alveolar ridges of the upper and lower jaws.

In the USA, according to the National Center for Health Statistics, in 2010, 4,392,000 bone and joint surgeries were made. Approximately 1 million of them involved cranial bones, extremities, ribs and sterna affected by injuries, postsurgical deformations, and oncological and inflammatory diseases, and 1,394,000 more were joint replacements of the lower extremities (with regard to revision surgeries). Bone grafting materials were required at least in 20–25% of the cases. There were 500,000 spine fusions (including 27,000 reoperations), which usually utilized bone substitutes, and 21,000 cases of arthrodesis [1]. In other words, the total number of surgeries using bone grafting materials was at least 1.3–1.5 million. As the total number of autogenic bone harvesting procedures did not exceed 207,000, the need for approved bone substitutes is evident.

Bone grafting is also required for one of every four dental implants [2]. According to the estimate of Straumann (Germany), the total number of implants annually placed all over the world is not less than 10.7 million [3]. The demand for bone substitutes is more than 2.5 million units in this category of indications alone.

More than 200 bone grafting materials have been approved for clinical use all over the world. A larger number of products are investigated in experimental and clinical studies. The variety of materials for bone grafting is the result of not only high demand but also the lack of a universal medical device that is effective in most clinical cases. Even with a correctly chosen treatment plan and an optimal surgical technique with advanced medical equipment, the bone substitute may often predetermine the unpredictability and, in some cases, unacceptability of the clinical outcome.

The variety of bone grafts that have been implemented in clinical practice and in various studies should be systematized. For this purpose, various material classifications based on nature, chemical composition, physical properties, and other parameters have been described [4]. Chronological classification has also been proposed to divide all developed bone substitutes into five generations: xeno-, allo-, and autogenic bone fragments not specifically processed; preserved allogenic bone materials; bone matrix analogues of synthetic and natural origin, including items with growth factors; tissue-engineered bone grafts; and gene-activated bone substitutes [5]. All of these systems are logical but have only theoretical relevance that is not associated with therapeutic indications and, accordingly, do not aid in the selection the most optimal variant of material in a particular clinical situation. Hereby, exactly applied classification is required that would combine both theoretical aspects significant for biomaterial specialists and practical aspects that physicians need. The review is intended to formulate and justify precisely such systematization.

## 2. Modern Trends in the Development of Bone Grafts

*The first technological trend* includes the majority of bone grafts approved for clinical applications that do not contain biologically active components standardized by qualitative and quantitative parameters. The present category, which may be referred to as “ordinary materials,” includes allogenic and xenogenic bone matrixes from various processing technologies (demineralized, deproteinized, etc.) [6]; calcium phosphates (*β*-tricalcium phosphate [7], octacalcium phosphate [8], etc.); natural or synthetic hydroxyapatite [9]; synthetic (PLGA, etc.) [10] and natural (collagen, chitosan) organic polymers [11]; silicates [12]; and composite products of the abovementioned materials.

It is well known that bone substitutes may possess different properties that have specific effects on reparative osteogenesis. Such properties include* osteoconduction, osteoprotection*,* osteoinduction,* and* osteogenicity* [13]. The majority of ordinary bone grafts have mainly osteoconduction. Some of them (e.g., demineralized bone matrixes derived using different processing technologies and calcium phosphates) are additionally characterized by a moderate osteoinductive effect, most likely due to optimal physical and chemical properties and (or) the presence of indefinite biologically active substances in the matrix that are not standardized by qualitative and qualitative parameters [14]. Their principal mechanism of action is to guide the bone regeneration, and their range of ultimate effectiveness is limited to the natural course of reparative osteogenesis that is appropriate for substitution of bone defects with high activity of native osteoinductive factors, but not enough for large bone defect repair.

It is established that large bone defects that present serious clinical problems are characterized by “osteogenic insufficiency.”* Osteogenic insufficiency* is a pathological condition associated with the low activity of systemic or local osteoinductive factors (Table 1) and (or) a low count of cambial cells in the bone lesion area, so that the natural process of reparative osteogenesis may not provide its complete histo- and organotypic recovery [15].

Causes of osteogenic insufficiency may be divided into local and general; the former includes defect size, geometry, number of walls [16], damaging factor (high- and low-energy injuries), presence of pathological inflammatory processes and related factors, and low density of functional blood vessels in the bone defect area, and the latter includes age [17], coexisting disorders (diabetes mellitus [18, 19], osteoporosis [20]), social habits (smoking) [21, 22], and administration of drug products that negatively affect osteogenesis (cytostatic agents [23] and possibly bisphosphonates [24], although a meta-analysis by Xue et al. (2014) did not show any negative effects of these drugs on fracture healing time [25]).

**Figure 1:**
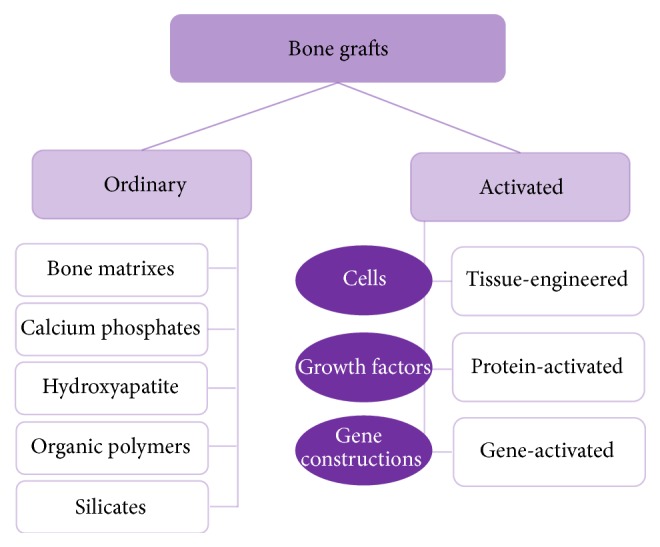
Generalized classification of current bone grafts.

It is therefore reasonable to divide all bone defects into two groups based on the absence/presence of osteogenic insufficiency. The first group is characterized by high activity of natural reparative processes, so optimization of bone regeneration alone is sufficient to decrease the treatment term and derive a larger volume of newly formed bone tissue. In the latter, defects with osteogenic insufficiency are determined by poor intensity of osteogenesis, and, accordingly, they require not optimization but rather induction and maintenance of reparative processes on a high level that may be achieved by introducing additional growth factors, substances increasing their synthesis, or cells that are able to produce them. In other words, ordinary materials are ineffective for the substitution of bone defects with osteogenic insufficiency, as they cannot modulate the effects of factors regulating osteogenesis. For that, ordinary materials as the scaffolds are combined with cells, growth factors, or gene constructions encoding them. The development of complex materials containing biologically active components presents the* second technological trend* that integrates “activated materials.” Based on the nature of osteoinductive components, the items may be divided into three main groups: tissue-engineered and protein- and gene-activated (Figure 1).

## 3. Activated Bone Grafts

### 3.1. Tissue-Engineered Bone Grafts

This group of materials includes items that contain two main components, a bioresorbable scaffold and live (auto- or allogenic) cells. The principal idea of the approach is to replace lost cambial reserves and increase the concentration of osteoinductive factors in the material implantation area. With high survival rates, cells transplanted into the recipient site may have a beneficial therapeutic effect due to two mechanisms of action: direct, differentiation to specialized cells of impaired tissues (indicated for autogenous cells [26]), and indirect, paracrine effect, the modulation of morphofunctional activity of other cells by the production of biologically active substances that are the factors of local osteogenesis regulation (Table 1). According to many authors, the paracrine activity of cells of the tissue-engineered bone graft, in particular, is their main mechanism of action [67]. Among the most significant factors for reparative osteogenesis produced by transplanted cells, bone morphogenetic protein (BMP), vascular endothelial growth factor (VEGF), and stromal-derived factor (SDF-1) should be specified. It is of interest that 10 million bone marrow multipotent mesenchymal stromal cells (MMSCs) in vitro produce approximately 750 pg/mL VEGF, 1030 pg/mL of TGF*β*1 [68], and 220 pg/mL of SDF-1 per day [69], whereas 10 million osteogenic periosteal cells secrete up to 40 ng/mL of BMP-2 and 200 pg/mL of VEGF per day [70].

#### 3.1.1. Bone Morphogenetic Proteins

BMP are members of the transforming growth factor family discovered in the second half of the 20th century whose biological effect is not limited by bone tissue. They are so referred to because they were first discovered in demineralized and lyophilized bone matrixes that were implanted into rabbit muscles and showed osteoinductive properties [71]. Among all members of the BMP family, BMPs 2, 4, 6, 7, and 9 have the largest impact on cells of osteoblastic differon (lowest, BMPs 3, 5, 8, and 10–15) [35].

**Figure 2:**
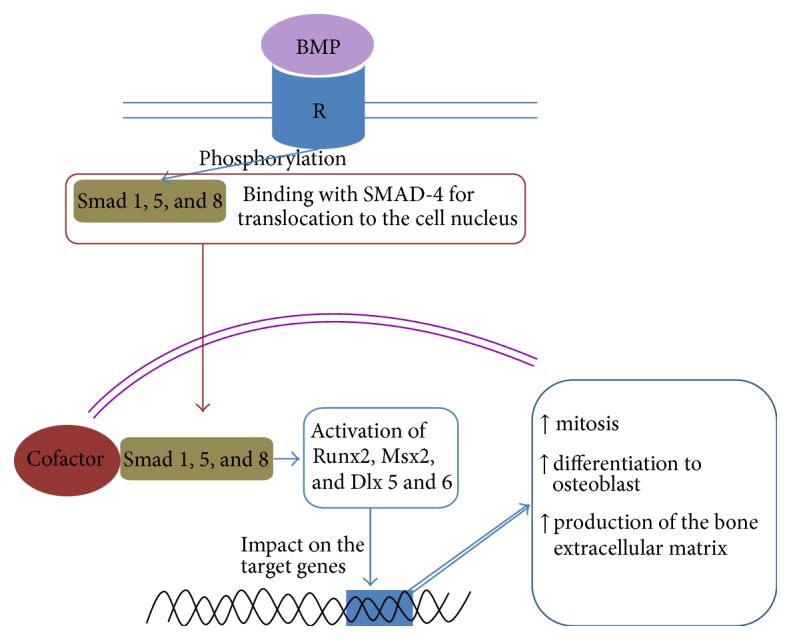
Scheme of intracellular Smad-mediated transduction pathway for BMP signals. BMP: bone morphogenetic protein; R: receptor.

Binding BMP with specific membrane tyrosine kinase receptors (types 1 and 2) results in the phosphorylation of intracellular proteins Smad-1, Smad-5, and Smad-8, which after activation form a “transport” complex, with Smad-4 translocating them to cell nuclei. In the nuclei, the Smad receptor proteins increase the expression of genes encoding key transcription factors responsible for the activation of the “osteoblastic phenotype” in cells (Figure 2) [72–77]. Such transcription factors include Runx2 (runt-related transcription factor 2) [77, 78], Msx2 [79], and Dlx 5 and 6 [80]. By interacting with each other and with other transcription factors, such as Osx (osterix) [81, 82], they affect target genes. As a result, they increase the proliferative activity of progenitor cells (mainly Msx2 [79]) and differentiation to osteoblasts, as well as the production of the components of the bone intracellular matrix (osteocalcin, bone sialoprotein, alkaline phosphatase, and collagens of types III and I) [81, 83]. Interestingly, two other Smad types, 6 and 7, have an inhibitory effect on Smad-mediated BMP action [84]. The intracellular Smad signal pathway is not solely for BMP [27], and the list of Smad activators and transcription factors is not limited to the transforming growth factor family (BMP and TGF-*β*).

Loss-of-function mutations in genes encoding BMP-2 or key intracellular proteins (Runx2, Msx2, Dxl 5 and 6, Osx, etc.) providing transduction of its signals result in the development of severe disorders that are nonsurvivable in homozygote status. Therefore, genetically mediated BMP-2 deficiency leads to increased bone fragility, disturbance of endochondral osteogenesis, and mineralization of the bone matrix [85, 86]. Hereby, only BMP-2 function could not be compensated by the activities of other proteins: the selective knockout of other BMPs (4, 7) does not have a significant effect on the histophysiology of skeletal bone, although it is accompanied by pathological symptoms from other organs and systems (urinary, cardiovascular, etc.) [87, 88]. Loss of function of the *α*-subunit of Runx2 due to mutation, if the identical *β*-subunit is preserved (Runx2^+/−^), results in the formation of cleidocranial dysplasia (dysostosis) [89, 90], whereas the Runx2^−/−^ genotype is nonsurvivable [91]. Autosomal-dominant craniosynostosis is based on Msx2 gene mutations [92].

#### 3.1.2. Vascular Endothelial Growth Factor

VEGF is a family of biologically active proteins first isolated by Folkman et al. in 1971 [93] that comprises the main auto- and paracrine regulation factors of vasculo-, angio- (VEGF A and B and PIGF), and lymphogenesis (VEGF C and D); they are produced by cells of all body tissues including epithelial.

**Figure 3:**
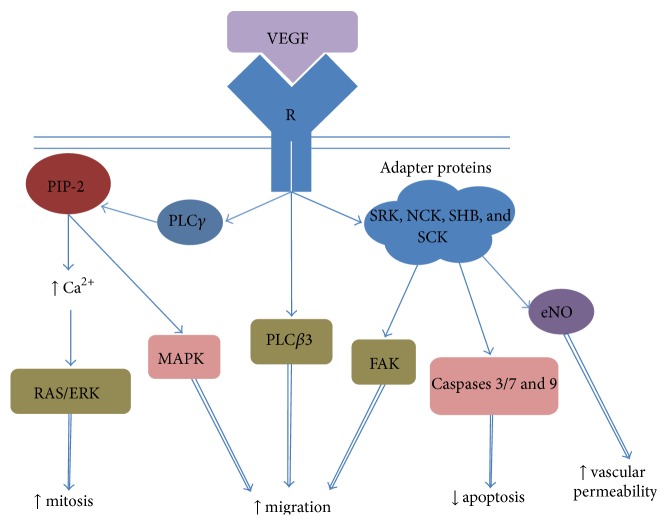
Scheme of the intracellular cascade pathway of VEGF signals. VEGF: vascular endothelial growth factor; R: receptor; PIP-2: phosphatidylinositol biphosphate; PLC*γ*: phospholipases С*γ*; PLC*β*: phospholipases C*β*; SRK, NCK, SHB, and SCK: group of adapter proteins; MAPK: mitogen-activated protein kinase; ERK: complex of extracellular-signal-regulated kinase; FAK: focal adhesion kinase; eNO: endothelial NO-synthase.

In postnatal period of human development, VEGF-A (isoforms 121, 145, 148, 165, 183, 189, and 206) [94] has the greatest impact on the formation of blood vessels. There are three types of VEGF receptors. Types 1 and 2 are involved in angiogenesis, and type 3 is involved in the formation of lymphatic vessels. Hereby, type 1 receptor has a greater affinity to VEGF, but its tyrosine kinase activity is much lower than that of type 2 receptor, which is considered one of the regulatory mechanisms preventing excessive VEGF activity. Correspondingly, VEGF effects are implemented via the type 2 receptor [43, 44]. After VEGF interaction with a specific type 2 receptor, the intracellular tyrosine sites of its kinase and carboxyterminal domains undergo autophosphorylation (Y951, 1054, 1059, 1175, and 1214) [44] which, in turn, activate several intracellular proteins such as phospholipases С*γ*, C*β*3, and adapter proteins SRK, NCK, SHB, and SCK, which are the first complex cascades of signal transduction that change the morphofunctional state of target cells (mainly endothelial). In particular, phospholipase С*γ* hydrolyses membrane phospholipid PIP_2_ by forming diacylglycerol and inositol-1,4,5-triphosphate, which increases the intracellular calcium levels that activate protein kinase С, which, in turn, initiates the subsequent activation of signalling pathway RAS-ERK leading to mitosis induction. As a result, the proliferative activity of endothelial cells is increased [43]. Phospholipase C*β*3 is involved in actin polymerization and the formation of stress-fibrils that provide migration and motor cell activity [45]. VEGF suppresses apoptosis via activation of the “phosphoinositide 3-kinase—protein kinase B (PI3K/AKT)” signalling pathway, inhibiting caspases 3, 7, and 9 and increasing cell survival. Moreover, axis PI3K/AKT along with calcium ions modulate the activity of endothelial NO-synthase, which is accompanied by a rise in NO production and an increase in vascular permeability, which leads to angiogenesis (Figure 3) [95, 96]. Thus, VEGF via a specific type 2 receptor induces activation, migration, proliferation, and differentiation of endotheliocytes and their progenitor cells, increasing cell survival, which, combined with the modulation of intracellular interactions and increase in vascular permeability, are essential prerequisites for the formation of capillary-like structures and subsequent remodelling into mature vessels [43–45, 95–98]. Because in both primary and secondary osteogenesis vessels sprouting into fibrous or cartilaginous tissues, respectively, provide the necessary conditions for the differentiation of resident cells into osteoblasts, as well as the migration of cambial reserves (perivascularly and with blood flow), VEGF-А may be considered an indirect osteoinductive factor.

Along with the angiogenesis-mediated effect, VEGF also has a direct influence on osteoblastic differon cells that not only produce VEGF [99] but also express its type 1 and 2 receptors both in embryogenesis [100] and the postnatal period of development [101]. It is shown that the proliferation of cambial cells of bone tissue exposed to VEGF significantly increases (up to 70%), and the migration of osteogenic cells is activated by the gradient of VEGF concentration [39–41].

More recently, apart from the canonical, a receptor, mechanism of VEGF action on the progenitor cells of osteoblastic differon, data on a fundamentally different “intracrine” mechanism is available. Its existence is confirmed by results showing that progenitor cells committed to osteoblasts (expressing Osx) synthesized VEGF not only for “export” but also to differentiate themselves to osteoblasts [102]. Liu et al. (2012) investigated bone marrow MMSC cultures obtained from healthy mice (control) and animals with a “loss-of-function” mutation of the gene encoding VEGF. It appeared that cells of the experimental group underwent osteogenic differentiation to a lesser extent than those of the control; hereby, their adipogenic potential was increased. The addition of recombinant VEGF to the culture medium of the “mutant” cells did not result in normalization of osteogenic differentiation, and the addition of antibodies blocking the VEGF receptors in the control was not accompanied by negative effects. However, after transfection of cells in the experimental group by a retroviral vector with the* vegf* gene to compensate for the knockout, an increase in the intracellular concentration of VEGF proteins was observed, which led to normalization of osteogenic differentiation and a simultaneous decrease of adipogenic potential [103].

Therefore, VEGF has a wide spectrum of action on cells of endothelial and mesenchymal cellular differons involved in reparative osteogenesis having both an angiogenesis-mediated stimulatory effect and a direct inducing impact on osteoblastic cells via the receptor and intracrine mechanisms.

#### 3.1.3. Stromal-Derived Factor-1

SDF-1 (CXCL12) is a protein from the chemokine group represented by two forms derived from alternative splicing, SDF-1*α* (89 amino acids) and SDF-1*β* (93 amino acids) [104]. Both are produced by cells of the bone marrow, fibroblastic and osteoblastic differons, and perivasculocytes.

**Figure 4:**
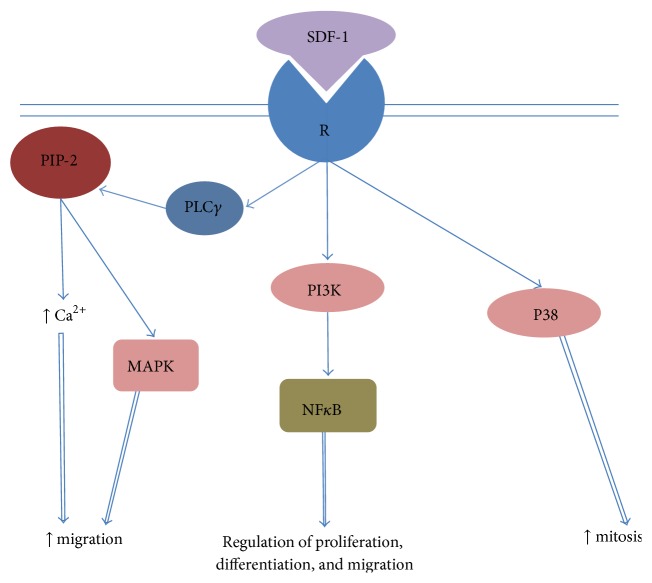
Scheme of intracellular cascade pathway of SDF-1 signal transduction. SDF-1: stromal-derived factor-1; R: receptor, PIP-2: phosphatidylinositol biphosphate; PLC*γ*: phospholipases С*γ*; MAPK: mitogen-activated protein kinase; NFkВ: nucleic factor-kappa В; PI3K: phosphoinositide-3-kinase.

The main SDF-1 receptor is CXCR4. After formation of a complex with the ligand, an intracellular G-protein that consists of three subunits (*α*, *β*, *γ*) is activated by separating into a heterodimer G*β*/*γ* and monomer G*α* (4 isoforms) possessing both different and common intracellular pathways of signal transduction. G*β*/*γ* activates phospholipase С*γ* which, as mentioned above, increases the release of calcium ions from intracellular depots and, via subsequent chains, activates MAPKs (mitogen-activated protein kinases) which, in turn, initiate chemotaxis (Figure 4) [105]. Moreover, the cell impulse to migration is also provided via PI3K activation, and p38 provides the impulse to proliferation [106]. Transcription factor NF-kappa В, whose level is increased under exposure to SDF-1, has a wide range of actions due to its increase of expression by more than 200 target genes encoding proteins involved in the regulation of cell proliferation, differentiation, and migration [107, 108]. It should be noted that recent data indicates that NF-kappa В, in general, inhibits osteogenesis via suppression of osteoblastic cambial cell differentiation. In this regard, SDF-1 secreted by osteoblasts from the lesion area [109, 110] may bring a bifacial effect: inducing the homing of progenitor cells including MMSC to the target area [46] and inhibiting their differentiation to osteoblasts. However, there are reasons to suppose that the effects, “undesirable” during a certain period of time, may be eliminated by other factors. In particular, it is shown that activated Smad proteins (1, 5) may inhibit SDF-1 production of osteoblasts [111]. Hence, in the inflammatory phase when activity of BMP proteins is reduced, osteoblasts actively secrete SDF-1 to attract additional cambial reserves, including endothelial precursors to the bone defect by a gradient of the chemokine concentration. With the transition of the recovery process to the phase of regeneration, with the increase of BMP-2 and BMP-7 levels and under their action (through Smad 1 and 5), bone cells cease producing SDF-1, which inhibits differentiation of the migrated progenitor cells to osteoblasts. During the late stages of regeneration and as part of the remodelling of newly formed bone tissue when cancellous bone should be populated with cells of bone marrow, the BMP level is decreased, whereas SDF-1 is again increased, which provides a homing of hematopoietic stem cells (HSC). The same mechanism allows cells of the osteoblastic line to hold HSC within bone marrow niches formed by both MMSCs and osteoblasts [112].

Cells for the development of tissue-engineered bone grafts may be either expanded by cultural technologies or used immediately as a “fresh-population” after harvesting from a tissue source. The main types of cells exposed to in vitro processing are MMSCs [113, 114], osteogenic cells, and osteoblasts [115, 116], as well as their combination. For that purpose, some investigators use endotheliocytes as independent [117] or additional cell components [118] and even induced pluripotent stem cells [119]. Uncultured cell populations include bone marrow cells (a mixture of MMSCs, fibroblasts, endothelial progenitor cells, HSC and definitive blood cells, etc.) [120] and the stromal-vascular fraction of adipose tissue (SVF-AT) (MMSCs, endotheliocytes and endothelial progenitor cells, smooth muscle cells, fibroblasts, preadipocytes, and immunocompetent cells) [121].

Multiple preclinical studies have shown the safety and efficacy of different variants of developed tissue-engineered bone grafts [114] that formed the basis for clinical translation. Moreover, several bone substitutes containing live cells have already been registered and approved for clinical application:

1. Allogenic: “Osteocel plus” (NuVasive, USA) (2005), “Trinity Evolution” (Orthofix, USA) (2009), “AlloStem” (AlloSource) (2011), “Cellentra VCBM” (BioMet, USA) (2012), and “OvationOS” (Osiris Therapeutics, USA) (2013).
2. Autogenous (“cell service,” which consists of harvesting a primary cell population, cultivation, combination with an appropriate scaffold, and transfer to the clinic for use): “BioSeed-Oral Bone” (BioTissue Technologies, Germany) (2001) and “Osteotransplant DENT” (co.don, Germany) (2006).

The large number of registered tissue-engineered bone grafts proves both the safety and the efficacy of the approach for certain bone defects. The majority of the registered products (Trinity Evolution, AlloStem, Osteocel Plus, Cellentra VCBM) are indicated for particular variants of spondylo- and arthrodesis, and others are for substitution of jaw defects (BioSeed-Oral Bone, Osteotransplant DENT). “Osteocel Plus” is one of the first and the most successful tissue-engineered bone grafts. The product is allogenous spongy bone tissue with live cells (5.25 × 10^5^  ± 4.6 × 10^3^ in 5 mL [122]) that are preserved in the composition due to a special “gentle” processing technology for cadaveric material with immunodepletion. Osteocel Plus is intended for spine surgery, including cervical spinal fusion. Separate clinical studies were performed on each of the five surgery types in which the product was indicated, a total number of 384 patients were enrolled, and adverse events were not reported. Most patients (over 90%) achieved complete fusion at five to six months after surgery [123]. The successful results of pilot clinical studies were published on the use of Osteocel Plus for other indications, such as augmentation of the alveolar ridge and arthrodesis of the lower extremities [124]. The developers associate the mechanism of action of Osteocel Plus and other allogenous tissue-engineered bone grafts with the osteoinductive effect of the matrix, as well as the paracrine activity of cells producing BMP, VEGF, SDF-1, and other growth factors [122]. A certain concentration of biologically active substances is also contained in the bone matrix.

None of these tissue-engineered bone substitutes has been registered in Russia as a medical product approved for clinical application, although successful results in the area have been obtained since the 1980s [125, 126]. This is mainly due to the absence of approved legal regulations on the registration of medical products consisting of cells. Nevertheless, pilot and initiative clinical studies under local authorities, beyond registration, are ongoing [127–130]. In particular, successful results were obtained with the use of tissue-engineered bone grafts consisting of autogenous adipose-derived MMSCs and two types of matrices (hydroxyapatite and a composite material of hydroxyapatite and collagen) in the treatment of patients with the atrophy of the alveolar bone of the upper jaw and the alveolar part of the lower jaw at the A.I. Evdokimov Moscow State University of Medicine and Dentistry [128]. A pilot clinical trial on the safety and efficacy of tissue-engineered bone graft of tricalcium phosphate and autogenous gingiva-derived MMSCs with sinus lifting has been initiated at the A.I. Burnazyan Federal Medical Biophysical Center (NCT02209311) [129].

However, several negative aspects of tissue-engineered bone grafts should be mentioned:

- lack of efficacy for large bone defects due to the death of most cells shortly after the transplantation of the tissue-engineered bone graft (cells require an active blood supply which is crucially minimized in a large lesion area) [130];
- high self-cost and complexity of technological process (cellular service) for making tissue-engineered bone grafts in accordance with GMP and GTP standards;
- impossibility to organize full-scale batch production of the most effective personalized (containing autogenous cells) tissue-engineered products;
- special storage conditions that are not always available at medical institutions (e.g., temperatures below −80°С);
- complexities of legal regulation and registration of medical products containing live cells.

Thus, the tissue-engineered approach to the development of activated bone substitutes allows the creation of safe medical products that are effective for certain indications. However, there are some problems that limit the implementation of tissue-engineered products to routine clinical practice that predetermines the development of alternative approaches.

### 3.2. Bone Grafts with Growth Factors

This group includes bone grafts consisting of a scaffold and growth factors (one or a few) that provide an osteoinductive effect; this is the most successful trend considering the precedents of clinical translation. Numerous products have already been registered and approved for clinical use such as “Emdogain” (Straumann, Germany), a material with enamel matrix proteins (1997); “OP-1” (Stryker Biotech, USA), with recombinant BMP-7 (2001); “Infuse” (Medtronic, USA) (2002, 2004, and 2007), with recombinant BMP-2; “GEM21S,” “augment bone graft” (BioMimetic Therapeutics Inc., USA), with recombinant PDGF-ВВ (2005, 2009); and “i-Factor Putty” (Cerapedalloics, USA), with protein P-15 (ligand for integrins *α*2*β*1 expressed by cells of an osteoblastic line) (2008).

“Infuse” was approved by the FDA for interbody spinal fusion in 2002, for bone grafting in shin bone fractures in 2004 (in combination with intramedullar fixation), and for sinus lifting and augmentation of the alveolar ridge in defects related to tooth extraction in 2007 [131]. The product is manufactured as a set consisting of a collagen matrix and recombinant BMP-2, which should be combined immediately prior to use. For spine surgery, because of the suboptimal biomechanical properties of the material, it should be implanted in a complex with special metallic cages. 270 patients were enrolled in the first clinical study and underwent anterior lumbar interbody spinal fusion. Of these patients, 143 had surgery with Infuse, and the others had surgery with a bone autograft of the iliac crest. During the two-year follow-up, adequate safety was shown, as well as high efficacy of treatment, with fusion rates of 94.5% and 88.7% in the clinical and control groups, respectively (the differences were not statistically significant). Only in patients of the control group (5.9%) were adverse events related to autograft withdrawal identified [132]. Subsequently, several postmarketing clinical studies were performed; the results were published, and a systematic analysis revealed the safety and efficacy of Infuse to be equal to those of bone autografts [133–135].

However, critical articles were also published that emphasized the complications and adverse events of Infuse, as well as their concealment by the company-developer [136, 137]. A special issue of the journal “Spine” was fully devoted to the problem, including the central review of the chief editor Carragee et al. (2011). The authors conducted a detailed analysis of 13 official clinical studies on Infuse, including reports submitted to FDA, on a total of 780 patients and revealed that the rates of complications and adverse events (osteolysis with horizontal or vertical implant dislocation, lack of fusion, retrograde ejection, heterotopic ossification, radiculitis, and infections) were approximately 10% for on-label application and up to 50% for thoracic or cervical spinal fusion [136]. Summarizing the results of multiple clinical studies on all three Infuse indications, it was stated that “on-label” use of the product is safe and effective in most cases, although there is a definite possibility of complications, as well as unsatisfactory results requiring resurgery. The off-label application, for other variants of bone grafting, is accompanied with a significant increase in the risk of complications and adverse events [137]. The total number of surgeries with bone substitutes containing BMP alone (mainly Infuse) in 2010 was 107,000, and the total number of spine interventions with interbody cages was 206,000 [1]. This shows both the success of bone grafts with growth factors and perhaps the high rates of off-label application of Infuse.

Some bone substitutes with growth factors are at different stages of experimental and clinical studies in Russia. For example, the results of the evaluation of bone grafts with recombinant BMP-2 [138] or VEGF [139] have been published.

Advanced studies on the development of bone substitutes with growth factors focus on two main aspects, the combination of several factors including angiogenic and osteogenic, for example, VEGF and BMP-2, in one scaffold [140] and providing the prolonged and controlled release of therapeutic proteins from the matrix structure, in particular due to the regulated dynamics of hydrogel matrix biodegradation [141–143] or the encapsulation of growth factors into microspheres made of organic polymers [144]. Some authors changed the structure of growth factors using special technologies (e.g., site-directed mutagenesis) to combine several factors, creating “mutant” molecules with higher efficacy in the activation of reparative osteogenesis. For example, Kasten et al. (2010) modified growth/differentiation factor-5 (GDF-5) by adding BMP-2 sites to its sequence to enable it to bind with specific receptors. As a result, molecule GDF-5 acquired properties typical of BMP-2 [145, 146].

Bone grafts with growth factors also have shortcomings and problems that limit their efficacy. Firstly, protein molecules in surgical wounds (due to exudation and the high activity of proteolytic enzymes) undergo rapid biodegradation, making them short-lived, which does not allow the bone substitute to demonstrate its osteoinductive action to the fullest extent. Second, the amount of therapeutic protein is limited, and its action is short-term and difficult even with controlled and limited release. In other words, the low concentration of protein molecules that left the scaffold and preserved its biological activity reaches a target cell, interacts with specific receptors on its surface, and induces a biological effect. Hereby, the receptors are rapidly inactivated in the presence of the ligand as compensatory adaptation mechanism that protects cells from excessive stimulation. The biological effect of growth factors will cease, and the protein concentration will be exhausted.

Theoretically, tissue-engineered and gene-activated materials are devoid of such shortcomings. In the first case, surviving cells protractedly produce a range of biologically active substances that accurately react on microenvironment signals, and, in the second one, they act more gently and are long-term compared to bone substitutes with growth factors, as therapeutic proteins are produced for certain period of time due to the expression of gene constructions delivered to target cells that can be regulated by the microenvironment.

### 3.3. Gene-Activated Bone Grafts

The main active component of these products is gene constructions (nucleic acids). In this regard, the development of gene-activated bone grafts is directly related to the advances of gene therapy, in which gene constructions are used as active substances of gene-therapeutic drugs.

Since 1989, more than 1900 clinical studies on gene therapy have been already registered [147], which highlights the activity of the conducted studies. Moreover, several gene-therapeutic drugs have been already implemented into routine clinical practice: “Gendicine,” “Oncorine” (SiBiono GeneTech, China), “Neovasculgen” (HSCI, Russia), and “Glybera” (uniQurо, Netherlands). “Neovasculgen” is a Russian research product approved for clinical use in Russia and Ukraine [148]. The experience of “Neovasculgen” development has been extrapolated to the first and currently only clinical trial of a gene-activated bone graft (NCT02293031).

A gene-activated bone substitute is a complex “scaffold-nucleic acid” combined using methods such as “chemical binding” [149], adjuvants (e.g., gel biopolymers) [150], or direct incorporation of nucleic acids into the scaffold at a certain stage of the matrix synthesis. The total efficacy of the product is thereby determined with the total mechanism of action including both gene construction (osteoinduction) and a scaffold (osteoconduction).

Two subsequent stages may be distinguished in the mechanism of the osteoinductive action of a gene-activated bone graft, nonspecific and specific. The first is associated with the release of nucleic acids from the scaffold structure after implantation into the bone defect area, delivery to the cells of recipient area and expression. This step is similar for any gene construction, and the variability of transfection is provided mainly by transgene delivery systems. The second consists of the specific action of a protein regulatory molecule produced by transfected cells which act as “bioreactors of therapeutic proteins,” synthesizing them for a certain period of time. In contrast to bone substitutes with growth factors, the main component of a gene-activated bone graft acts “gently,” as mentioned above. In other words, transgene entry into the nucleus of target cell does not force obligatory expression of the therapeutic protein. The cell preserves its normal functional state and reaction to microenvironment stimuli, so that if the therapeutic protein is not needed at a particular period in time, the transfected cell may decrease the mRNA of the transgene by intracellular posttranscriptional mechanism regulated stability and the half-life of mRNA and thereby prevent protein production [151]. This mode of action of gene constructions significantly increases the efficacy of gene-activated bone grafts in comparison with substitutes containing growth factors [152].

Gene constructions consist of a therapeutic gene (cDNA or RNA) and its intracellular delivery system (vector). Vectors are divided into two main groups, viral and nonviral. In the first case, a transgene is incorporated into a particle of retro-, lenti-, and adenovirus or adenoassociated virus, and, in the second case, a transgene is incorporated into a plasmid, a circular molecule of nucleic acids containing several additional sequences providing transgene expression. Viral and nonviral delivery systems differ in their efficacy of transfection. 40% or more of viral gene constructions can enter target cells, and the rate of plasmid DNA uptake (“naked”) does not exceed 1-2% due to its size and negative charge. Some approaches were proposed (physical and chemical) to increase the efficacy of plasmid DNA transfection up to 8–10% [153].

It should also be mentioned that several viral vectors (retro- and lentiviral, etc.) are incorporated into the genome. In other words, a transgene has an almost lifelong expression, and others, including plasmid DNA, are not integrated into the genome and therefore only temporarily expressed for 10–14 days. Considering that the production of a therapeutic protein encoded by a gene construction should not exceed the terms of complete reparative regeneration, retro- and lentiviral vectors are rarely used in making a gene-activated bone graft; they are more often applied in the gene-cellular approach, wherein a cell culture is transfected ex vivo and then combined with a scaffold [154].

Hence, all gene-activated materials may be divided by the technology of scaffold and gene construction combinations, as well as by the compositions of their biologically active components: the nature of the vector or transgenes or the number of transgenes or various gene constructions in one product. However, it is evident that the main differences in the biological effect of a gene-activated bone graft are driven by the transgene. Nucleotide sequences encoding the main osteoinductive and osteoblast-specific transcription factors are, as expected, the most frequently used for the development of gene-activated bone substitutes (Table 2).

Among the transgenes most often selected for induction of recovery processes are* bmp*, especially* bmp-2* (Table 2), and* vegf*. The first studies were related to direct gene transfer; the method injected gene constructions into the soft tissues surrounding the bone defect as a solution, that is, without immobilization on or into a scaffold [155, 156]. It is important that, even in such a case, positive results were obtained, which proved the supreme importance of gene constructions in gene-activated materials. In particular, in the study by Baltzer et al. [155], complete consolidation was shown at 12 weeks after adenovirus administration (2 × 10^10^ particles) of the DNA encoding gene* bmp-2* in the muscle around defects (1.3 cm) of the femur in rabbits. In the control, in which a gene encoding a fluorescent marker protein (luciferase) without osteoinductive activity was used as a transgene, a central part of the defect was preserved in all cases and filled by fibrous tissue. Until now, in vitro or in vivo direct gene transfer was mainly used in bone indications for the selective assessment of the biological effect of gene constructions chosen for the development of gene-activated bone grafts. Feichtinger (2014) et al. developed a coexpressive plasmid DNA with genes encoding BMP-2 and BMP-7 that is subcutaneous injected as a solution (20 *μ*g) and found that, in 46% of cases, induction of heterotopic osteogenesis resulted [196].

However, despite the published positive results of direct gene transfer, without mechanical filling of the bone defects with osteoconductive materials, especially in cases of large defects, complete recovery of the bone seems impossible. In this regard, gene-activated bone grafts have become the logical “evolution” of direct gene transfer. The peak for the development of such products containing gene constructions with* bmp* occurred in 2004–2007, which may be related to the prior success of an alternative approach: the FDA approval and wide use in clinical practice of bone substitutes containing growth factors BMP-7 (OP-1, Stryker Biotech, USA) and BMP-2 (Infuse, Medtronic, USA) in 2001 and 2002, respectively.

Subsequently, the specification of the role of angiogenesis in bone regeneration, as well as a detailed description of the intracellular signal pathways regulating proliferation, differentiation, and the morphofunctional activity of bone cells, formed a fundamental ground for an increasing number of investigators to use sequences encoding VEGF as transgenes and some transcription factors as well (Table 2).

Keeney et al. (2010) developed a gene-activated bone graft made of a collagen-calcium-phosphate matrix and plasmid DNA encoding VEGF-А165 (0.35 *μ*g/mm^3^ of the carrier). The item was implanted subcutaneously in mice and into the defects of the intercondyloid fossa of the femur (diameter 1 mm, length 7 mm). Although no signs of osteogenesis or a significant difference in the number of vessels appeared under heterotopic conditions, a significantly larger volume of bone was regenerated in the experimental group under orthotopic conditions than in the control (a scaffold with DNA encoding a marker gene) at 30 days after surgery [209]. However, the experimental model for the assessment of bone graft efficacy could not be considered optimal due to minimum size of the defect.

In Russia, some variants of gene-activated bone substitutes have been already developed using* vegf-a* as a transgene and different scaffolds (xenogenic bone matrix, composite material of collagen and hydroxyapatite, octacalcium phosphate, etc.). The efficacy of the products was shown in a more complex model, with the substitution of bilateral cranial defects (diameter 10 mm) of parietal bones in rabbits [149, 210].

Based on an analysis of the published study results associated with development of gene-activated bone grafts (as well as the gene-cellular approach and direct gene transfer), we can conclude that most of them showed acceptable safety and high effectiveness in the experimental models, regardless of the vector type and scaffold. However, some difficulties remain for gene-activated materials in general: manufacture, sterilization, standardization of control for preservation of the specific activity of the gene construction after the completion of the production cycle, and the necessity of increasing the transfection level of nonviral gene constructions and enabling their prolonged release from the scaffold's structure after implantation.

## 4. Conclusions

A detailed understanding of the regulation features of reparative osteogenesis, its dynamics, and results depending on the presence or absence of osteogenic insufficiency, as well as a comprehension of the modes of action characterized for various groups of bone grafts that fall under two main technological trends, allows us to reconsider the modern system of bone substitutes and propose a new classification with their division into two groups: “*ordinary*” and “*activated*” materials.

The category of ordinary materials includes items that do not contain biologically active components standardized by qualitative and quantitative parameters. Osteoconduction and, in some cases, moderate osteoinduction allow these materials to optimize reparative regeneration for the promotion and increase in size of newly formed bone tissue. They are therefore intended for substitution of bone defects (and recovery of jaw atrophy) in the absence of osteogenic insufficiency. The main problem for this category of bone substitutes is low osteoinductive potential, which surgeons often mitigate using an improvised, empirical activation of mixing the material with the patient's blood, autogenous bone (generally, in a ratio of 1 : 1), plasma enriched with thrombocytes, or plasma enriched with growth factors immediately prior to implantation [211].

Due to their biologically active components, activated materials have pronounced osteoinduction and (or) osteogenicity and are therefore able to both support the natural course of reparative osteogenesis and induce and provide high activity up to complete histotypical recovery. This quality makes them theoretically applicable for substitution of even large bone defects that are characterized by osteogenic insufficiency. Autogenous bone tissue is a prototype, a type of reference sample or a “gold standard” of materials for bone substitution [212]. The origin of their development lies in the necessity to develop effective alternatives for autogenous bone that may allow limiting or completely eliminating the use thereof.

The complex composition and mode of action of activated bone grafts predetermine the necessity to carry out more comprehensive standardized preclinical studies with individual assessment of active components (growth factors, cells, or gene constructions) in terms of safety and biological action.

The practical value of the proposed classification of all bone substitutes as ordinary and activated, along with developing an understanding of osteogenic insufficiency, is to form a fundamental ground for physicians to make the most effective objective choice of bone graft for every particular clinical situation. However, before using the presented system, some additional studies should be performed on methods for the quantitative evaluation of osteogenic insufficiency and the real clinical efficacy of all variants of activated bone grafts.

## Figures and Tables

**Table 1:** The main factors of the local regulation of reparative osteogenesis. EPC: endothelial progenitor cells.

Factor	Effect on osteogenesis	Effect on angiogenesis
BMP-2, BMP-4	Activation of proliferation, differentiation, synthesis of components of bone intercellular matrix, and growth factors (VEGF, bFGF, etc.) [27, 28] Biological action is decreased by impact of BMP-3 [29].	Influence on EPC. Stimulation of migration, proliferation, and formation of capillary-like structures; increase of VEGF and ANG-1 receptor expression; no effect on cell differentiation and survival [30, 31].

BMP-3	Suppression of differentiation; decrease of osteogenic activity [29].	—

BMP-6	Decrease of proliferative activity of MMSCs and activation of their differentiation [32] (to a greater extent than the other BMPs [33]).	Activation of EPC proliferation; organization of capillary-like structures [34].

BMP-7	Activation of proliferation, differentiation, and synthesis of components of bone intercellular matrix [35].	Increase of endothelial cell proliferation, production of VEGF receptors, and induction of capillary-like structure formation [36].

BMP-9	Increase of bone intercellular matrix production without negative regulation by BMP-3 [37].	Activation of endothelial cell proliferation, including production of angiogenic factor receptors (VEGF and ANG-1) [38].

Vascular endothelial growth factor (VEGF)	Increase of proliferative activity, differentiation, and chemotaxis induction by gradient of concentration [39–42].	Stimulation of proliferation, differentiation, migration, formation of capillary-like structures, and inhibition of endothelial cell apoptosis [43–45].

Stromal-derived factor-1 (SDF-1)	Induction of cambial cell homing by concentration gradient and inhibition of differentiation [46].	Activation of migration, proliferation, adhesion, and differentiation of EPCs [47].

Angiopoietins 1 and 2	—	Activation of differentiation; intercellular contact formation of endothelial cells in vessel wall (vascular stabilization) [48, 49].

Erythropoietin	Stimulation of MMSC differentiation to osteoblasts and monocytes to osteoclasts, without increase of their activity [50]; increase of chondrocyte proliferation [51].	Stimulation of endothelial cell proliferation [52] and NO production [53].

Basic fibroblast growth factor	Increase of proliferation and suppression of differentiation [54].	Increase of proliferation and suppression of EPC differentiation [55].

Hepatocyte growth factor	activation of differentiation and synthesis of bone intercellular matrix components [56].	Activation of proliferation and migration [57], inhibition of apoptosis, and decrease of endothelial permeability [58].

Insulin-like growth factor-1	Increase of mechanic sensitivity of specialized cells, induction of differentiation, and synthesis of bone intercellular matrix components in response to physical exercise [59].	Activation of migration, proliferation and differentiation of endothelial cells, and induction of capillary-like structure formation [60].

PDGF-AA	Insignificant increase of proliferation and differentiation; chemotaxis activation (to lesser extent than when exposed to PDGF-BB) [61]; increase of IGF-1 production.	—

PDGF-BB	Activation of cell proliferation and migration [62].	Induction of pericyte migration, adhesion and incorporation to walls of forming vessels, and activation of EPC migration [63].

TGF-*β*1	Increase of proliferative activity, decrease of differentiation, and synthesis of bone intercellular matrix components [64].	Activation, migration, proliferation, and formation of capillary-like structures [65].

Angiogenin	—	Release of endothelial cells from vascular vessels and their activation and stimulation of migration and proliferation [66].

**Table 2:** Compositions of gene constructions developed for induction of reparative osteogenesis (as components of gene-activated bone grafts or gene-cellular products).

Number	Transgene	Vector	References
*Genes encoding growth factors/hormones*			

1	Angiopoietin-1		[157]

2	BMP-2	Plasmid DNA	[156]
		Adenoviral	[155]
		Lentiviral	[158]
		Liposomal	[159]

3	BMP-4	Plasmid DNA	[160]
		Retroviral	[154]

4	BMP-6	Plasmid DNA	[161]
		Adenoviral	[162]
		Adenoassociated	[163]
		Lentiviral	[161]

5	BMP-7 (OP-1)	Plasmid DNA	[164]
		Adenoviral	[165]
		Adenoassociated	[166]
		Retroviral	[167]

6	BMP-9	Plasmid DNA	[168]
		Adenoviral	[169]

7	BMP-12	Plasmid DNA	[170]

8	Cyclooxygenase-2 (Cox-2)	Retroviral	[171]

9	Erythropoietin (EPO)	Adenoviral	[172]

10	Epidermal Growth Factor (EGF)	Plasmid DNA	[173]

11	bFGF	Plasmid DNA	[174]

12	HGF	Adenoviral	[175]

13	HIF-1*α*	Lentiviral	[176]

14	IGF-1	Plasmid DNA	[177]

15	Integrin-*α*5	Lentiviral	[178]

16	LIM mineralization protein-1 (LMP-1)	Retroviral	[179]

17	LMP-3	Adenoviral	[180]

18	Nell-1	Adenoviral	[181]

19	Osterix	Retroviral	[182]

20	PDGF-А	Adenoviral	[183]

21	PDGF-B	Plasmid DNA	[184]
		Adenoviral	[183]

22	Parathyroid hormone (amino acids 1–34)	Plasmid DNA	[185]

23	TGF-*β*1	Nonviral vector (K)16GRGDSPC	[186]

24	VEGF-А	Plasmid DNA	[187]
		Adenoviral	[188]

25	BMP-2 + BMP-7	Adenoviral	[189]

26	BMP-2 + BMP-6	Adenoviral	[190]

27	BMP-2 + IHH	Adenoviral	[191]

28	BMP-2 + VEGF	Adenoviral	[192]

29	BMP-2 + VEGF + IGF-1 + TGF-*β*1		[193]

30	BMP-7 + PDGF-b	Adenoviral	[194]

31	RANKL + VEGF	Adenoassociated	[195]

32	BMP-2/BMP-7	Plasmid DNA	[196]

33	BMP-2/BMP-4	Liposomal	[197]

34	BMP-6/BMP-9	Adenoviral	[198]

35	BMP-6/VEGF	Adenoviral	[199]

36	BMP-7/IGF-1	Adenoviral	[200]

37	BMP-7/OPG	Plasmid DNA	[201]

*Genes encoding transcriptional factors*			

38	Cbfa1	Lentiviral	[202]
		Adenoviral	[203]

39	c-myb	Plasmid DNA	[204]

40	Runx2	Adenoviral	[205]
		Retroviral	[206]

41	SOX9	Adenoassociated	[207]

42	caALK6 + Runx2	Plasmid DNA	[208]
